# Surfactant-Switched Positive/Negative Electrorheological Effect in Tungsten Oxide Suspensions

**DOI:** 10.3390/molecules24183348

**Published:** 2019-09-14

**Authors:** Alexander V. Agafonov, Anton S. Kraev, Tatiana V. Kusova, Olga L. Evdokimova, Olga S. Ivanova, Alexander E. Baranchikov, Taisiya O. Shekunova, Sergey A. Kozyukhin

**Affiliations:** 1 Krestov Institute of Solution Chemistry of the Russian Academy of Sciences, Ivanovo 153045, Russia; ava@isc-ras.ru (A.V.A.);; 2 Kurnakov Institute of General and Inorganic Chemistry of the Russian Academy of Sciences, Moscow 119991, Russia

**Keywords:** electrorheology, tungsten oxide, positive electrorheological effect, negative electrorheological effect, electroconvection

## Abstract

The electrorheological (ER) effect was experimentally observed in dielectric suspensions containing tungsten oxide (WO_3_) modified with surfactant molecules (sodium dodecyl sulfate (SDS) and dodecylamine (DDA)) in electric fields up to several kilovolts per millimeter. The dielectric properties of WO_3_ suspensions in silicone oil were analyzed, depending on the frequency of the electric field, in the range from 25 to 10^6^ Hz. Unmodified WO_3_ suspensions, as well as suspensions modified with sodium dodecyl sulfate, were shown to exhibit a positive electrorheological effect, whereas suspensions modified with dodecylamine demonstrated a negative electrorheological effect. The quantitative characteristics of the negative electrorheological effect in the strain–compression and shear regimes were obtained for the first time. Visualization experiments were performed to see the chain structures formed by WO_3_ particles modified with sodium dodecyl sulfate, as well as for dynamic electroconvection in electrorheological fluids containing WO_3_ modified with dodecylamine. The negative electrorheological effect was shown to be associated with the processes of phase separation in the electric field, which led to a multiplicative effect and a strong electroconvection of the suspension at field strengths above 1 kV/mm.

## 1. Introduction

The development of stimuli-responsive materials capable of changing their physical properties in response to external factors such as temperature, pH, light, mechanical loads, and electromagnetic fields is of considerable interest, both to applied and to fundamental science [1,2,3,4,5,6,7]. A special place among such materials is occupied by liquid systems that reversibly change their physical and mechanical properties in magnetic and electric fields—namely, magnetic, magnetorheological, and electrorheological (ER) fluids [8,9,10,11].

Electrorheological fluids are colloid systems in which a dielectric liquid (for example, silicone oil) acts as a dispersion medium and fine particles of oxides (or metal salts) serve as a dispersed phase [12]. The preparation of electrorheological fluids, which reversibly increase viscosity in the electric field, has recently received considerable attention due to the wide possibilities for the design of electrically controlled devices based on the electrorheological effect. Some aspects of the practical application of electrorheological fluids have been discussed in the reviews [12,13,14,15]. Most devices of practical interest are based on a positive electrorheological effect, such as ER clutches, brakes, damping devices, hydraulic valves, shock absorbers, robotic controlling systems, gripping devices, seismic controlling frame structures, human muscle stimulators, and spacecraft deployment dampers [12]. Other promising fields of electrorheological fluids and electrorheological effect application include the design of automated gauges for tactile recognition (e.g., shape of objects), the development of programmable surfaces for tactile sensors, and the creation of power and tactile displays. ER-based MEMICA (mechanical mirroring using controlled stiffness and actuators) power devices with feedback also attract considerable interest. Control mechanisms for aviation, vehicles, and computer games (power joysticks) and new types of stepper motors combining piezoelectric and electrorheological effects are being developed. Microdampers and micropumps for microelectromechanical devices are being designed [11,13,16].

There is still a significant demand for materials demonstrating a negative electrorheological effect and reversibly decreasing their viscosity in the applied electric field. The areas of practical application for the negative electrorheological effect are currently not very well developed, due to insufficiently clear knowledge about the origins of this effect. The production of a hydrostatic bearing on a negative electrorheological effect has been reported for the control of rotor vibration (negative ER hydrostatic journal bearing) [17]. Additionally, the utilization of a negative electrorheological effect for the separation of biomolecules has been proposed [18]. An unusual “colloidal motor” has been demonstrated recently by Lemaire and Lobry [19]. In that work, a drastic decrease in apparent viscosity (down to zero) was achieved by the electric field-induced rotation of insulating particles in a weakly conducting liquid medium.

Currently, several models have been proposed for the interpretation of the electrorheological effect, each of them considering different types of polarization of particles in electric fields [11,12,13,20,21,22,23]. The most well-known mechanisms for the origin of the electrorheological effect take into account electrostatic polarization [24,25], or formation of aqueous bridges between the particles as a result of polarization of adsorbed water molecules [26], or interphase (Maxwell–Wagner) polarization [27]. These models consider the presence of polar molecules on the surface of dispersed phase particles, the dielectric constant and the dielectric loss tangent of the particles, the differences in the conductivity of the dispersed phase, and the dispersion medium. Finally, the rate of dielectric relaxation of the particles has a certain effect as well.

It should be noted that the reasons for the development of the electrorheological effect have not yet been fully studied. For example, the polarization model underlying many theories describing the electrorheological effect is not able to explain the fact that some materials with high dielectric permittivity do not show electrorheological activity or exhibit only a weak electrorheological effect. Thus, a suspension of barium titanate, which has a dielectric constant of approximately 2000, does not exhibit an electrorheological effect in a direct electric field [28]. An even more significant problem is the explanation of the negative electrorheological effect, in which the yield strength and viscoplastic characteristics of the electrorheological fluid decrease with increasing electric field strength [29,30]. For some systems, the negative electrorheological effect has been shown to arise if the polarization characteristics of the liquid medium are higher than the polarization characteristics of the filler particle material [31]. Thus, the existing polarization models possess a number of limitations, and, for their further development, an extensive study of different ER filler materials (including the use of various surface modificators) is required, including the materials with unusual dielectric and physical properties. Special attention should be paid to a negative electrorheological effect observed under tangential stress, which is not only perpendicular but also parallel to the electric field applied. This will support new theoretical approaches for the interpretation of a negative electrorheological effect at the microscopic scale.

The physical properties of tungsten oxide make it a promising material for the design of electrorheological fluids [32,33]. In particular, this material has an extremely high dielectric permittivity (*ε*′) of 10^5^ (at 500 Hz) [34]. Notably, low-density materials are most commonly used as dispersed phase in ER fluids, which significantly reduces the role of the gravitational factor in the stability of suspensions [20]. There have been no investigations of the specific aspects of the interaction with electric fields of filler particles in electrorheological fluids based on high-density oxide particles (WO_3_ density is up to 7.4 g/cm^3^). The design of electrorheological fluids containing high-density particles requires additional efforts to maintain high sedimentation stability of the suspensions, which can be provided by surface modification of such particles with surfactants.

In the present study, an analysis of the electrorheological effect was conducted in novel WO_3_-based ER fluids, modified by various surfactant molecules (sodium dodecyl sulfate (SDS) and dodecylamine (DDA)), in tension, compression, and shear modes in electric fields up to several kilovolts per millimeter. Measurements of dielectric characteristics were carried out (frequency dependences of the dielectric constant and the dielectric loss tangent in the range from 25 to 10^6^ Hz) for ER fluids containing WO_3_ and silicone oil. Surprisingly, we found that different surfactants can switch the electrorheological effect to a positive or a negative value. Specifically, WO_3_ suspensions in silicone oil modified with sodium dodecyl sulfate were shown to exhibit a positive electrorheological effect, whereas suspensions modified with dodecylamine demonstrated a negative electrorheological effect. Thus, the use of different surface modificators in WO_3_ suspensions resulted in completely different electrorheological behavior.

## 2. Results and Discussion

According to X-ray diffraction data, the powder obtained by precipitation from an aqueous solution of ammonium tungstate, followed by thermal treatment at 100 °C, as well as products of its modification with SDS and DDA, was a single-phase crystalline tungsten oxide (monoclinic γ-WO_3_, space group P21/n, *a* = 7.303(1) Å, *b* = 7.532(1) Å, *c* = 7.686(1) Å, β = 90.56(2)°) (Figure S1, Supplementary Materials). This polymorph of tungsten oxide is known to be thermodynamically stable at room temperature [35]. The estimation of the particle size of tungsten oxide by Scherrer’s formula resulted in 75–80 nm values for all the samples.

The dilute suspensions of unmodified WO_3_ powder in silicone oil had low stability and were prone to segregation. At a concentration of 60 wt%, these suspensions were more stable (presumably due to the steric effect); however, mixing was occasionally required during the measurements. The WO_3_/SDS powder formed highly stable suspensions at high concentrations of the dispersed phase (40%–60% by weight). The WO_3_/DDA powder dispersed excellently in silicone oil, affording stable suspensions which, in the concentrated (60 wt%) state, demonstrated a toothpaste thickness. Figure S2 (Supplementary Materials) shows the sedimentation curves, illustrating the stability of 10 wt% suspensions of WO_3_, WO_3_/SDS, and WO_3_/DDA in silicone oil. Strength characteristics of electrorheological suspensions under tension and compression, as well as shear stress and dynamic viscosity outside the electric field, decreased in the series WO_3_/DDA > WO_3_/SDS > WO_3_.

**Figure 1:**
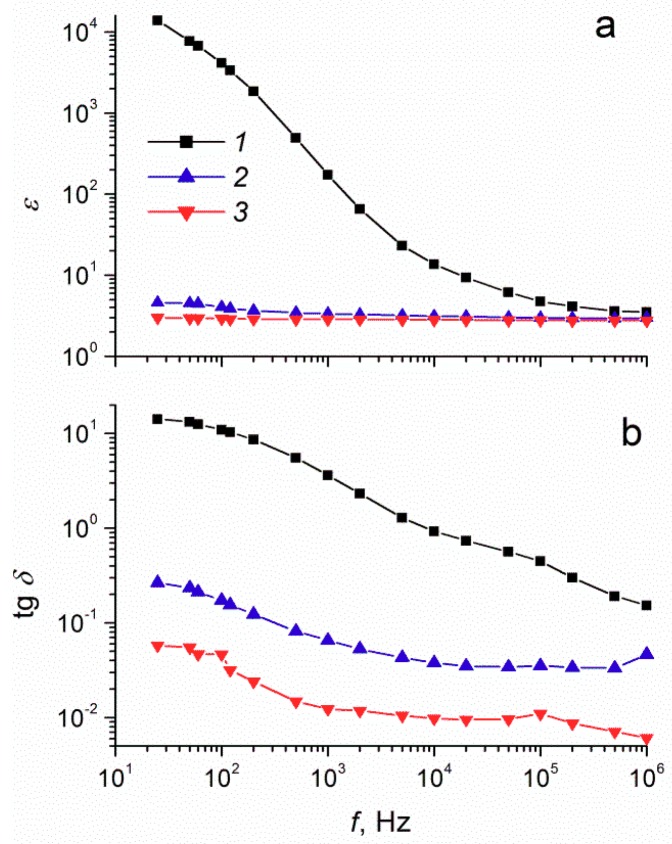
Frequency dependences of (**a**) the dielectric constant and (**b**) the dielectric loss tangent for 40 wt% suspensions of tungsten oxide (WO_3)_ (1), WO_3_/sodium dodecyl sulfate (SDS) (2), and WO_3_/dodecylamine (DDA) (3).

The dielectric spectra analysis for suspensions of modified and unmodified tungsten oxide powders with 40 wt% filler concentration (see Figure 1a,b) revealed that the dependences of the dielectric characteristics (*ε* and tg*δ*) on frequency significantly differed for ER fluids with different fillers. For ER fluids based on unmodified WO_3_, a significant increase in *ε* and tg*δ* was observed with a decrease in the frequency of the electric field. At 25 Hz, the dielectric constant was 14,000, and the dielectric loss tangent was 14.4. Such dependences are typical for crystalline tungsten oxide, for which dielectric permittivity increases with decreasing electric field frequency to 10^5^ at 500 Hz [34]. For surfactant-modified tungsten oxide suspensions, the values of the dielectric characteristics in the low-frequency region were much lower. In the region of high electric field frequency, the dielectric characteristics of suspensions with different fillers were nearly the same.

**Figure 2:**
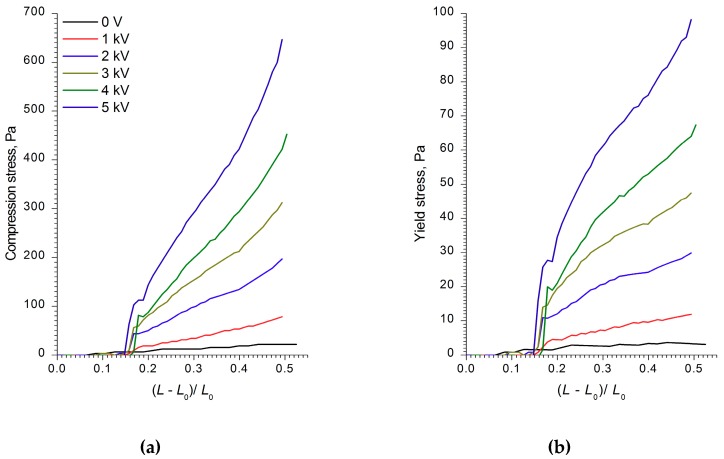
The dependences of (**a**) the compression stress and (**b**) the yield stress of electrorheological (ER) fluid with 60 wt% WO_3_ on the deformation degree $\frac{\left(L-L_{0}\right)}{L_{0}}$ at various values of voltage applied. The initial gap between the electrodes was 2 mm.

**Figure 3:**
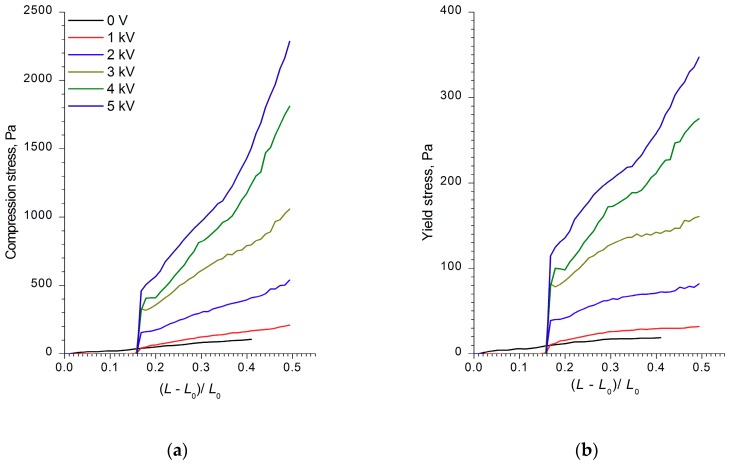
The dependences of (**a**) the compression stress and (**b**) the yield stress of ER fluid with 60 wt% WO_3_/SDS on the deformation degree $\frac{\left(L-L_{0}\right)}{L_{0}}$ at various values of voltage applied. The initial gap between the electrodes was 2 mm.

**Figure 4:**
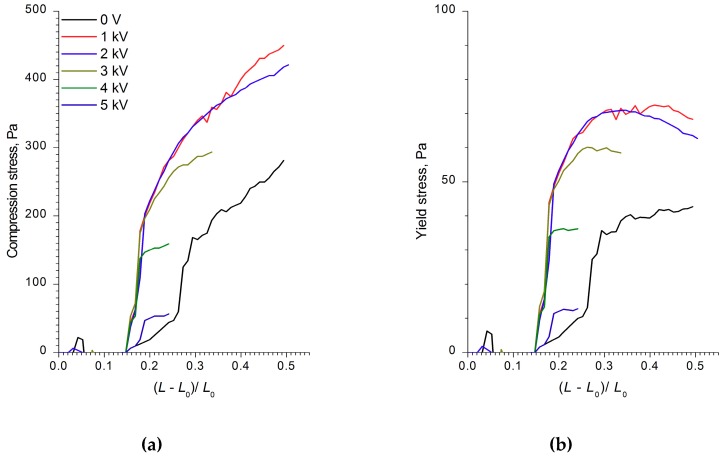
The dependences of (**a**) the compression stress and (**b**) the yield stress of ER fluid with 60 wt% WO_3_/DDA on the deformation degree $\frac{\left(L-L_{0}\right)}{L_{0}}$ at various values of voltage applied. The initial gap between the electrodes was 2 mm.

The analysis of the strain, compression, and shear stress dependences on the electric field strength, as well as the ER fluids’ strain values and shear rates, showed that the WO_3_, WO_3_/SDS, and WO_3_/DDA suspensions interacted differently with the electric field. Figure 2, Figure 3 and Figure 4 show the compression curves of electrorheological fluids containing WO_3_, WO_3_/SDS, and WO_3_/DDA for different values of the electric field strength applied. At low compression rates, the compressive stress *P* can be related to the yield point of the plastic fluid *τ*_0_ by the following equation [36,37], $P=\frac{D}{3h}\tau_{0}$, where *D* is the electrode diameter and *h* is the distance between electrodes. Based on the data regarding the effect of the electric field on the ER fluids’ compressive stress, we calculated the values of the yield stresses for ER fluids under compression. As one can note from Figure 2, Figure 3 and Figure 4, during compression (deformation degree is given as $\frac{\left(L-L_{0}\right)}{L_{0}}$), electrorheological fluids containing WO_3_, WO_3_/SDS, and WO_3_/DDA demonstrated different behavior. Upon compression in an electric field, suspensions of WO_3_ and WO_3_/SDS exhibited classical behavior. The strength of a fluid with WO_3_ in an electric field under compression was almost four times lower than that of a fluid containing WO_3_/SDS. As the field strength and compression ratio increased, the compressive stress increased (Figure 2 and Figure 3), with the yield stress for both compressed fluids approximately six times lower than the compression stress.

**Figure 5:**
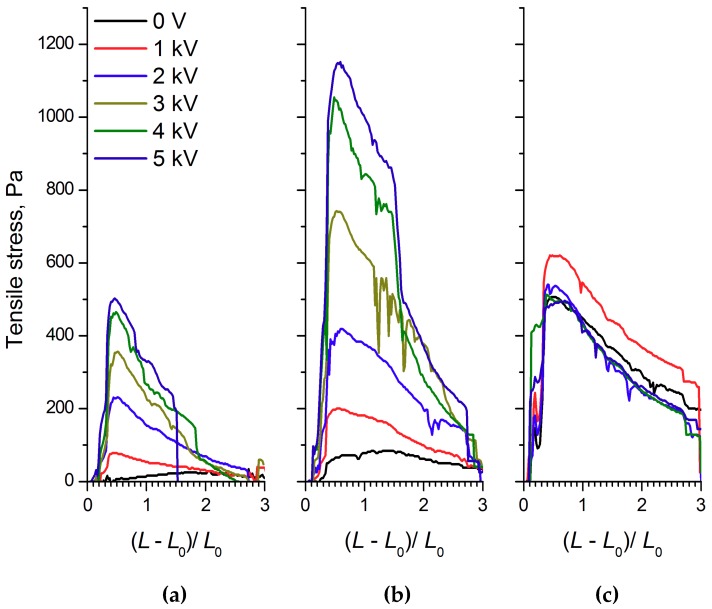
The dependence of the tensile stress of ER fluids with 60 wt% of (**a**) WO_3_, (**b**) WO_3_/SDS, and (**c**) WO_3_/DDA on the deformation degree $\frac{\left(L-L_{0}\right)}{L_{0}}$ at various values of the initial voltage applied. The initial gap between the electrodes was 1 mm.

**Figure 6:**
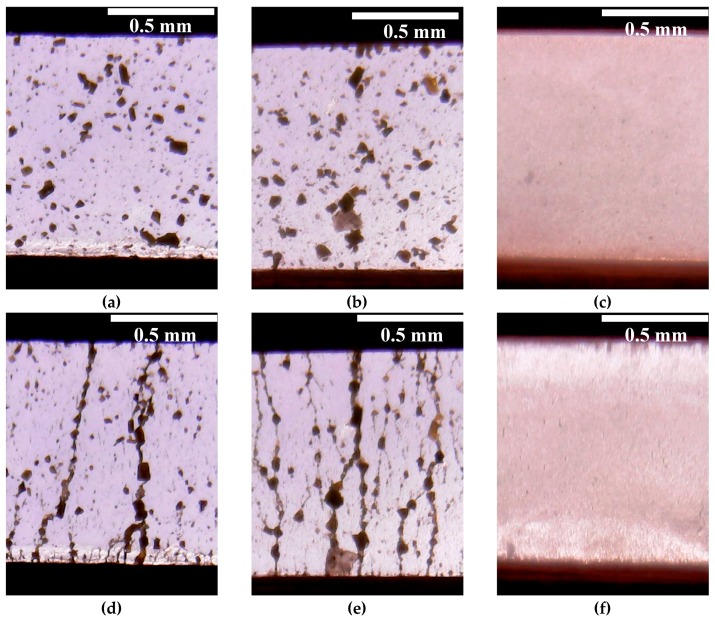
Experimental visualization of structure formation in the electrorheological fluids containing 5 wt% of (**a**,**d**) WO_3_, (**b**,**e**) WO_3_/SDS, and (**c**,**f**) WO_3_/DDA, using optical microscopy. Suspensions appeared (**a**–**c**) in the absence of an electric field and (**d**–**f**) in a 1 kV/mm electric field. In the microphotographs, the positive electrode is at the top.

The analysis of the shape of the strain curves for ER fluids based on WO_3_ and WO_3_/SDS (Figure 5a,b) revealed that these materials, in an electric field, behave in a similar manner. The maximum on the stretch curve characterizing the yield stress in the WO_3_-based ER fluid was observed at a degree of deformation of approximately 0.5, whereas for the WO_3_/SDS-based ER fluid the maximum appeared at a degree of deformation of approximately 0.75. Note that the yield stress in the electric field of the WO_3_/SDS-based ER fluid exceeded that of the WO_3_-based ER fluid more than 2-fold. Apparently, the observed differences were related to the effect of polar molecules, adsorbed on the filler particles, on the electrorheological effect. The increase of the electrorheological effect in the ER fluid with the surfactant-modified filler was in good agreement with previously published results [21,38,39]. According to microscopic observations, the electrorheological effect in WO_3_ and WO_3_/SDS suspensions in the electric field was accompanied by the formation of chain structures from the particles of the dispersed phase (Figure 6).

Unexpectedly, unlike the WO_3_- and WO_3_/SDS-based ER fluids, the ER fluid with WO_3_/DDA filler in the electric field demonstrated a negative electrorheological effect (Figure 5c). This means that we, for the first time, measured the quantitative characteristics of the negative electrorheological effect in the strain–compression and shear regimes.

Due to the uncommon behavior of the ER fluid containing WO_3_/DDA in the electric field, it is viable to interpret the observed physico-mechanical effects with consideration of direct visual microscopic monitoring of the structural changes taking place in the liquid when electric fields of different strength are applied. Visual monitoring of ER fluids containing 5% and 60% WO_3_/DDA showed that, in the electric fields, the structure formation in these systems proceeded in different ways.

For a dilute suspension (Figure 6f), as the field strength increased from 0 to 1 kV/mm, the WO_3_/DDA particles started migrating towards the nearest electrodes and settled on their surface, in the form of layers and short chains, in accordance with the phase separation model [28]. The depletion of the near-electrode layers of the dispersed phase led to the shielding of the electrodes and the formation of a layer of unstructured suspension of the dispersed phase particles in the interelectrode gaps (“clouds”).

**Figure 7:**
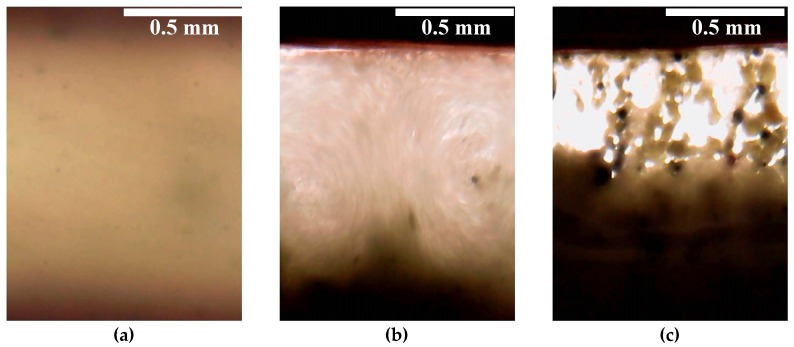
Experimental visualization of structure formation in electrorheological fluids containing 60 wt% WO_3_/DDA, using optical microscopy. Suspensions appeared (**a**) in the absence of an electric field, (**b**) in a 1 kV/mm electric field, and (**c**) in a 1.5 kV/mm electric field. In the microphotographs, the positive electrode is at the top.

The change in the structure of the concentrated 60 wt% WO_3_/DDA suspension with increasing field strength occurred according to a different scheme. In the range of voltages up to 1 kV/mm, the motion of the filler particles was observed, stimulated by the potential difference and the electric current (video file is available under Supplementary Materials). When a field strength of 1 kV/mm was achieved in the interelectrode gap, two electrohydrodynamic convective vortices were formed in the suspension, circulating between the electrodes. One of them rotated clockwise and the other rotated counter-clockwise (Figure 7). The mass transfer was directed from the cathode to the anode. Large aggregates of particles appeared in the structurally homogeneous suspension and participated in the electroconvection. Such an electroconvective motion was observed during the entire period of the electric field application. A similar effect was described earlier by Ramos-Tejada et al. with respect to a goethite suspension [30]. When a voltage of 1.5 kV/mm was applied to the cell gap with 60% WO_3_/DDA suspension, the particulate matter migrated to the negatively charged electrode. The particles were concentrated near the negative electrode, with some of the particles settling on the electrode and some remaining in the volume of the suspension. In this case, the suspension split into layers, with different concentrations of the solid phase, and large inhomogeneities arose from the aggregates of the filler particles (Figure 7c).

The structural transformations described affected the physico-mechanical characteristics of the ER fluids. As can be seen from Figure 5c, the tensile strength of 60% WO_3_/DDA ER fluid in an applied 1 kV/mm electric field exceeded the analogous characteristic of the ER fluid in a zero field. With a field strength of more than 1 kV/mm, the tensile strength of the fluid became practically even, with the tensile strength of the fluid stretched outside the field. Thus, an electroconvective transfer at a field strength of 1 kV/mm led to an increase in the tensile strength of the ER fluid.

**Figure 8:**
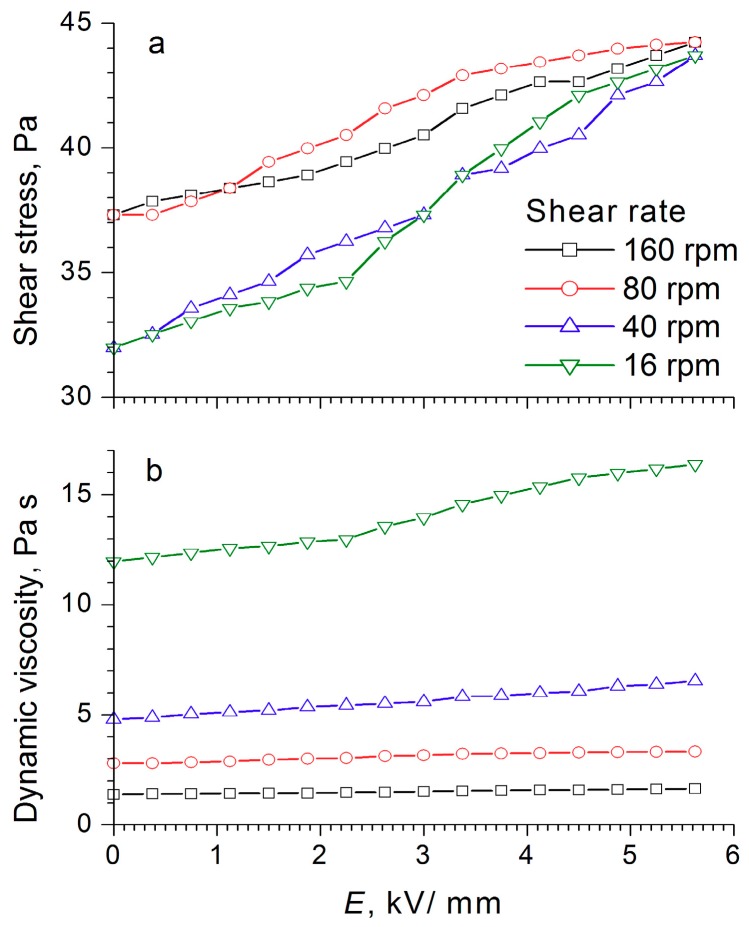
Dependence of (**a**) shear stress and (**b**) dynamic viscosity of the electrorheological fluid containing 60 wt% WO_3_ on the electric field strength, at different shear rates.

**Figure 9:**
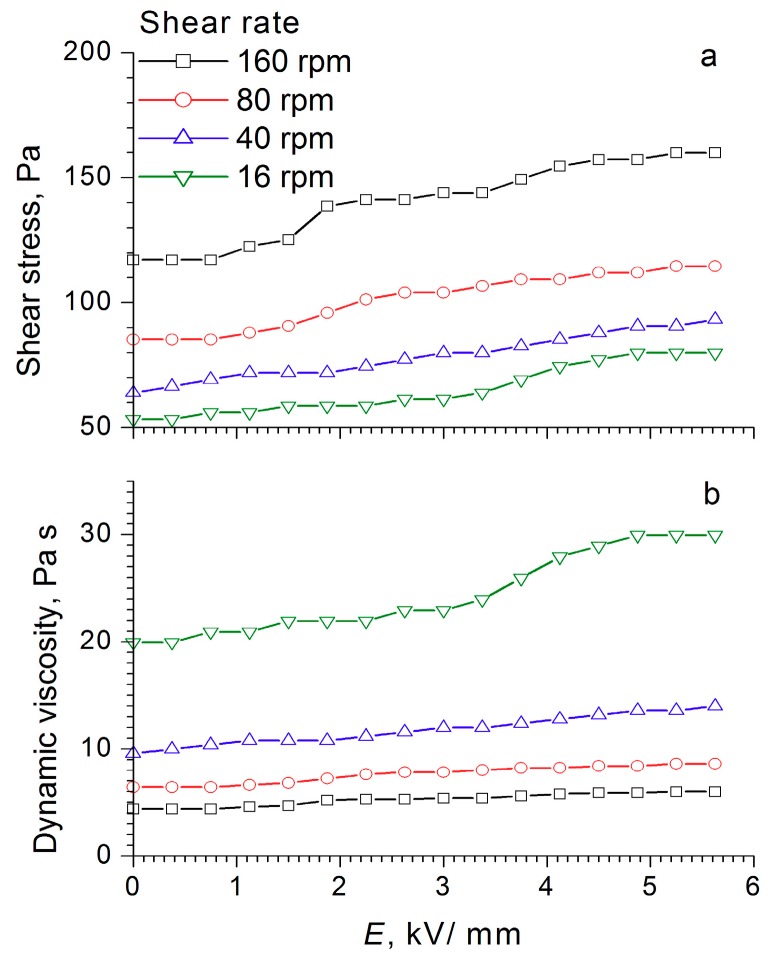
Dependence of (**a**) shear stress and (**b**) dynamic viscosity of the electrorheological fluid containing 60 wt% WO_3_/SDS on the electric field strength, at different shear rates.

**Figure 10:**
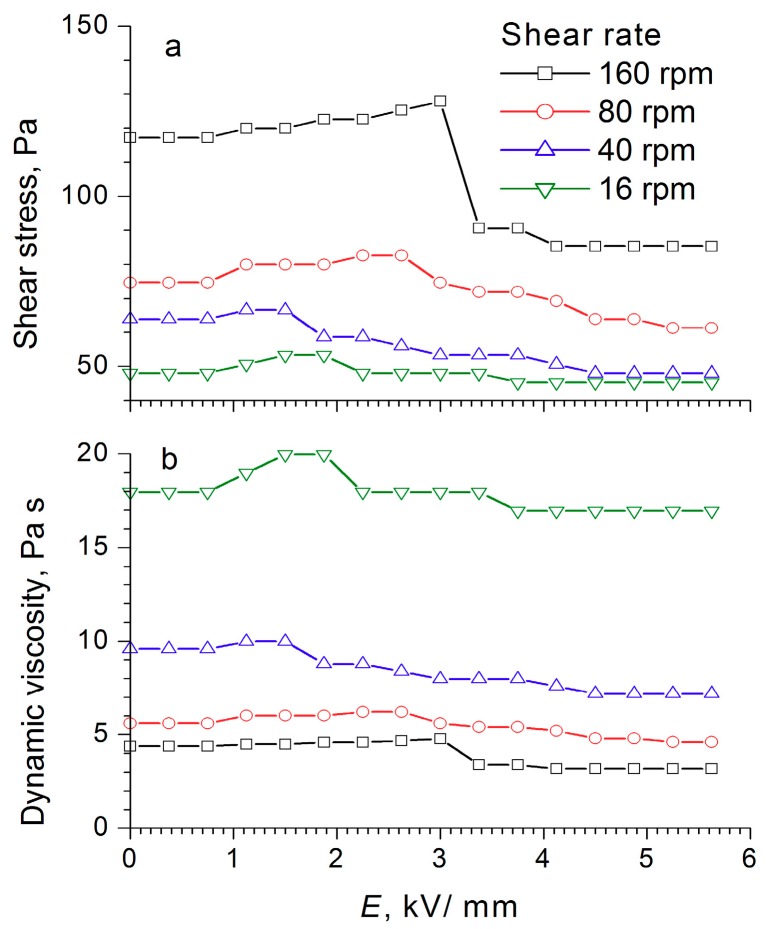
Dependence of (**a**) shear stress and (**b**) dynamic viscosity of the electrorheological fluid containing 60 wt% WO_3_/DDA on the electric field strength, at different shear rates.

During the compression of an electrorheological fluid with 60 wt% WO_3_/DDA (Figure 4) in the 1 kV/mm and 2 kV/mm electric fields, an increase of ER fluid strength was observed in comparison to the fluid with no electric field. At high values of the electric field strength, there was a regular decrease in compression stress and yield stress at compression (Figure 4). It is interesting to note that, in an electric field with 5 kV/mm strength, the strength of the ER fluid at compression was practically the same as the strength of PMS 300 silicone oil. The results of rheological studies (Figure 8, Figure 9 and Figure 10) indicate that the dynamic viscosity of 60 wt% ER fluid decreased with increasing shear rate and electric field strength.

For the electrorheological fluid containing 60 wt% WO_3_/DDA, two ranges could be observed in the graph of shear stress dependence on the field strength at different shear rates, and these were associated with the rearrangement of the structure of the fluid in an electric field (Figure 10). With increasing field strength after a certain critical value, the shear stress decreased (like the dynamic viscosity), the critical value of the field strength being dependent on the shear rate. For low shear rates, the threshold value laid in the region of low strengths, and as the shear rate increased, it shifted toward higher stresses. This was most clearly manifested at a shear rate of 160 rpm, when a sharp drop occurred in the shear stress—from 120 to 90 Pa at an electric field strength of 3 kV/mm—and a “step” was formed after which the shear stress became practically independent of the field strength values. The observed effects indicated a change in the structure of the WO_3_/DDA-based electrorheological fluid in response to the electric field applied, leading to a decrease in the mechanical properties of the suspension after reaching a certain threshold value of electric field strength, which depended on the shear rate. Direct microscopic observations of the behavior of the electrorheological suspension exhibiting a negative electrorheological effect in the electric field showed that, as the tension increased, the static structure of the suspension broke down and the transition to dynamic electroconvection occurred. This led to a nonlinear dependence of the properties of the ER fluid on field strength, direction, and rate of loading. In the general case, with a negative electrorheological effect, the strength characteristics of the suspension in the electric field decreased after reaching a critical strength in comparison with the strength of the suspension outside the electric field. These findings are consistent with the results obtained earlier [20,28,40,41,42].

We discovered that a change in the electrorheological effect from positive to negative can occur with a change in the type of adsorbed surfactant on the surface of solid inorganic filler particles. We believe that a negative electrorheological effect in an electrorheological fluid with a WO_3_/DDA filler can originate due to partial desorption of dodecylamine molecules from the surface of tungsten oxide particles induced by electric fields with strengths higher than 1 kV/mm. The desorbed dodecylamine molecules in the electric field form circulating electroconvection currents between the electrodes, involving the filler particles, which leads to an intensification of the electroconvective process, and changes in the structure and rheological characteristics of the ER fluid in the electric field. The electroconvection of dodecylamine molecules in an electric field was indicated by the results of direct visual observation of the same process in the dispersion of pure dodecylamine (in the absence of WO_3_) in silicone oil. It is interesting to note that electroconvection was not observed in the dispersion of pure sodium dodecyl sulfate in silicone oil.

## 3. Materials and Methods

To prepare tungsten oxide powders, an aqueous solution of 7 M hydrochloric acid (analytical grade) was added dropwise to 100 mL of a 0.1M aqueous solution of Na_2_WO_4_ (≥99% Sigma-Aldrich # 14304, Darmstadt, Germany) up to pH = 2, with vigorous stirring at room temperature. The resulting precipitate of tungstic acid was separated by centrifugation, washed with distilled water, and dried at 100 °C overnight. The corresponding sample was referred to as WO_3_ for this study. The modification of WO_3_ powder with sodium dodecyl sulfate (SDS) (≥99.0% Sigma-Aldrich # L6026) and dodecylamine (DDA) (98% Sigma-Aldrich #D222208) was performed by suspending the corresponding surfactants in reagent-grade isopropanol solutions, with vigorous stirring for 24 h at room temperature. The weight ratio of surfactant:WO_3_ in suspensions was 0.05. Afterward, isopropanol was removed from the suspensions by drying at 80 °C overnight. Tungsten oxide powders modified with SDS and DDA were further referred to as WO_3_/SDS and WO_3_/DDA, respectively.

The electrorheological fluids were prepared by mixing the filler powders (modified or unmodified tungsten oxide) with polydimethylsiloxane PMS-300 (PENTA Silicone, Moscow, Russia), followed by thorough grinding of the mixtures in an agate mortar until homogeneous suspensions were obtained. The filler content in the suspensions was 40 wt% or 60 wt%.

Measurements of shear stress as a function of the electric field strength and shear rate were carried out with a modified rotational CR-rheometer RN-211 (Rheotest Medingen GmbH, Ottendorf-Okrilla, Germany) with a controlled shear rate, equipped with a measuring system including parallel polished brass plates with a diameter of 20 mm and a gap of 1 mm between the upper sliding contact and a lower stationary electrode. The torque measuring error did not exceed 5%. Measurements of the tension and compression stresses in the electrorheological fluids at various electric field strengths were carried out on a computer-controlled press with a precision screw, whose plunger was moved by a stepping motor. The moving speed of the mobile electrode was 0.003 mm/sec. The load cell, with a sensitivity of 0.001 g, was automatically recorded at a frequency of 1 s^−1^. The electric field strength in the gap between the electrodes at tension or compression was calculated from the equation E = U/h, where U is the applied voltage and h is the current gap value in mm between the electrodes. The initial width of the gap in the tension measurements was 1 mm, whereas in compression measurements it was 2 mm. The detailed procedures for measuring the rheological characteristics of the suspensions in the electric field have been previously reported [43,44].

The dielectric measurements were carried out in a cylindrical cell of a capacitor type with polished stainless steel electrodes. Measurements of the frequency dependences of the dielectric constant and the dielectric loss tangent for electrorheological fluids were carried out on a Solartron SI 1260 Impedance/Gain-Phase analyzer (Solartron, Farnborough, United Kingdom).

To enable visualization of the electrorheological experiment, a cell was used that was made from a slide with copper electrodes pasted on it with a parallel gap of 1 mm. The cell was equipped with a video camera that allowed real-time video recording with a 40× magnification.

X-ray powder diffraction measurements were performed using a Bruker D8 Advance diffractometer (Bragg–Brentano geometry, Karlsruhe, Germany) with CuKα radiation.

All measurements were carried out at room temperature.

## 4. Conclusions

In this article, the first experimental evidence of the electrorheological effect in a tungsten oxide-based ER fluid is reported. Quantitative characteristics of viscoplastic properties for electrorheological fluids containing tungsten oxide fillers, including filler particles modified with surfactants, were obtained in strain, compression, and shear tests in electric fields up to 5 kV/mm in polydimethylsiloxane PMS-300. An increase in the strength of the applied electric field led to an increase in the strength characteristics of the abovementioned ER fluids, namely, shear stress, compressive stress, and strain tension.

A negative electrorheological effect was discovered in suspensions of tungsten oxide modified with dodecylamine, leading to a decrease in the strength of the suspension in the electric field in comparison to the viscoplastic properties of suspensions outside an electric field. The direct microscopic observations of the WO_3_/DDA suspension with a negative electrorheological effect revealed that, as the electric field strength increased, the static structure of the suspension was destroyed and the transition to the dynamic electroconvection regime occurred. The electroconvection could be due to the adsorption of polarized particles of the dispersed phase on electrodes with the formation of island structures, which led to an inhomogeneity of the electric field in the gap between the electrodes and the torque formation on the polarized filler particles. A visual observation of the electroconvection phenomenon was carried out for suspensions of tungsten oxide with different concentrations of the dispersed phase. In dilute suspensions, as the field strength increased, the particles of the dispersed phase did not form chains connecting the electrodes, but migrated towards the nearest electrodes and settled on their surface as layers and short chains. As a result, channels enriched with silicone oil and depleted in the dispersed phase were formed in the layers of fluid closest to the electrodes. An area of unstructured suspension was formed in the center of the interelectrode gap. In concentrated suspensions, when an electric field was applied, two electrohydrodynamic convective zones were formed in which the circulation of particles occurred in different directions. This led to a nonlinear dependence of the properties of the ER fluid on field strength and the direction and rate of load application. In general, in the electric field, the decrease in strength characteristics of the suspension occurred after a critical stress was achieved in comparison to the strength of the suspension outside an electric field.

## Supplementary Materials

The following are available online at https://www.mdpi.com/1420-3049/24/18/3348/s1, Figure S1: X-ray diffraction patterns of (**a**) WO_3_; (**b**) WO_3_/DDA; and (**c**) WO_3_/SDS; Figure S2: Sedimentation curves for 10 wt% suspensions of (1) WO_3_, (2) WO_3_/SDS, and (3) WO_3_/DDA powders in PMS-300 silicone oil; Video S1: Electroconvection of 60 wt% WO_3_/DDA electrorheological fluid in a 1 kV/mm electric field. Video was captured using an optical microscope in the gap between the electrodes (1 mm).
