# Supplementary material for: Surfactant-Switched Positive/Negative Electrorheological Effect in Tungsten Oxide Suspensions
Source: Molecules. 2019 Sep 14;24(18):3348. doi: 10.3390/molecules24183348 (PMC6767292; doi:10.3390/molecules24183348)
Supplement: Supplementary file 1 [file molecules-24-03348-s001.zip › Agafonov_ESI_revised.docx]

**Electronic Supplementary Information**

Surfactant-switched positive/negative electrorheological effect in tungsten oxide suspensions

Alexander V. Agafonov ^1^, Anton S. Kraev ^1^, Tatiana V. Kusova ^1^, Olga L. Evdokimova ^1^, Olga S. Ivanova ^2^, Alexander E. Baranchikov ^2,*^, Taisia O. Shekunova ^2^, Sergey A. Kozyukhin ^2^

^1^ *Krestov Institute of Solution Chemistry of the Russian Academy of Sciences*; ava@isc-ras.ru

^2^ *Kurnakov Institute of General and Inorganic Chemistry of the Russian Academy of Sciences*; a.baranchikov@yandex.ru

***** Correspondence: a.baranchikov@yandex.ru





Fig. S1. X-ray diffraction patterns of (a) WO_3_; (b) WO_3_/DDA; (c) WO_3_/SDS.





Fig. S2. Sedimentation curves for 10 wt.% suspensions of (*1*) WO_3_, (*2*) WO_3_/SDS and (*3*) WO_3_/DDA powders in PMS-300 silicone oil.
