# Supplementary material for: Surfactant-Switched Positive/Negative Electrorheological Effect in Tungsten Oxide Suspensions
Source: Molecules. 2019 Sep 14;24(18):3348. doi: 10.3390/molecules24183348 (PMC6767292; doi:10.3390/molecules24183348)
Supplement: Supplementary file 1 [file molecules-24-03348-s001.zip › Agafonov_ESI_revised.pdf]

Electronic Supplementary Information

Surfactant-switched positive/negative electrorheological effect in tungsten oxide suspensions

Alexander V. Agafonov [1], Anton S. Kraev [1], Tatiana V. Kusova [1], Olga L. Evdokimova [1], Olga S. Ivanova [2], Alexander E. Baranchikov [2,*], Taisia O. Shekunova [2], Sergey A. Kozyukhin [2]

[1] *Krestov Institute of Solution Chemistry of the Russian Academy of Sciences*; ava@isc-ras.ru
[2] *Kurnakov Institute of General and Inorganic Chemistry of the Russian Academy of Sciences*; a.baranchikov@yandex.ru
* Correspondence: a.baranchikov@yandex.ru

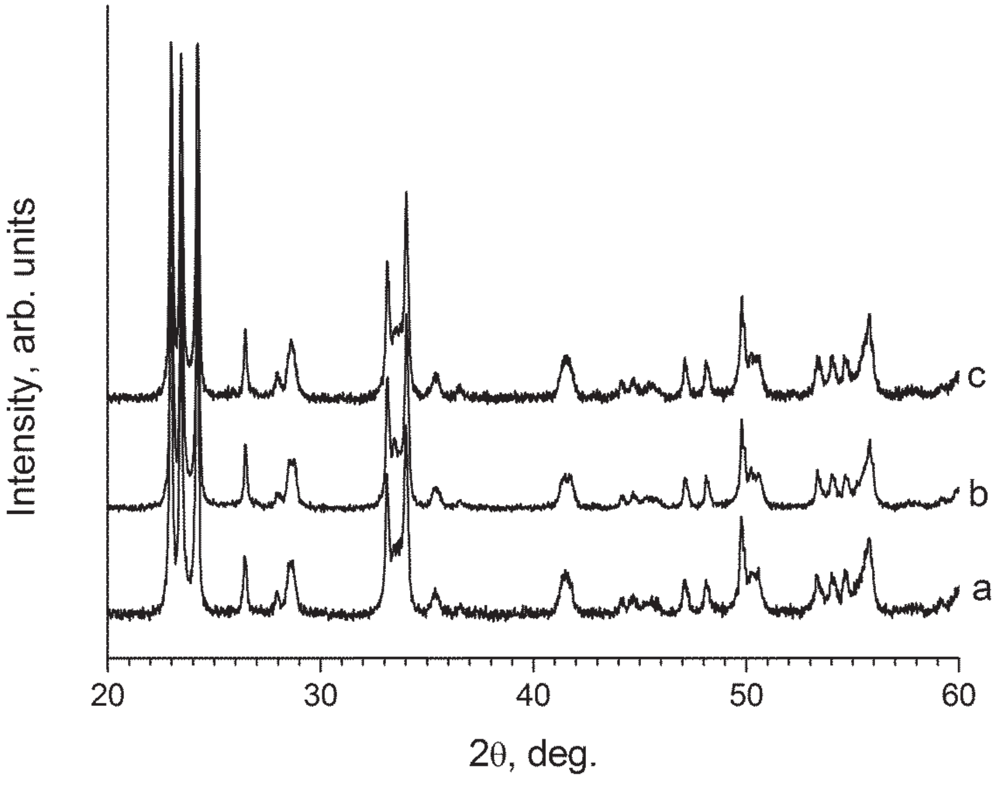

Fig. S1. X-ray diffraction patterns of (a) WO_3; (b) WO_3/DDA; (c) WO_3/SDS.

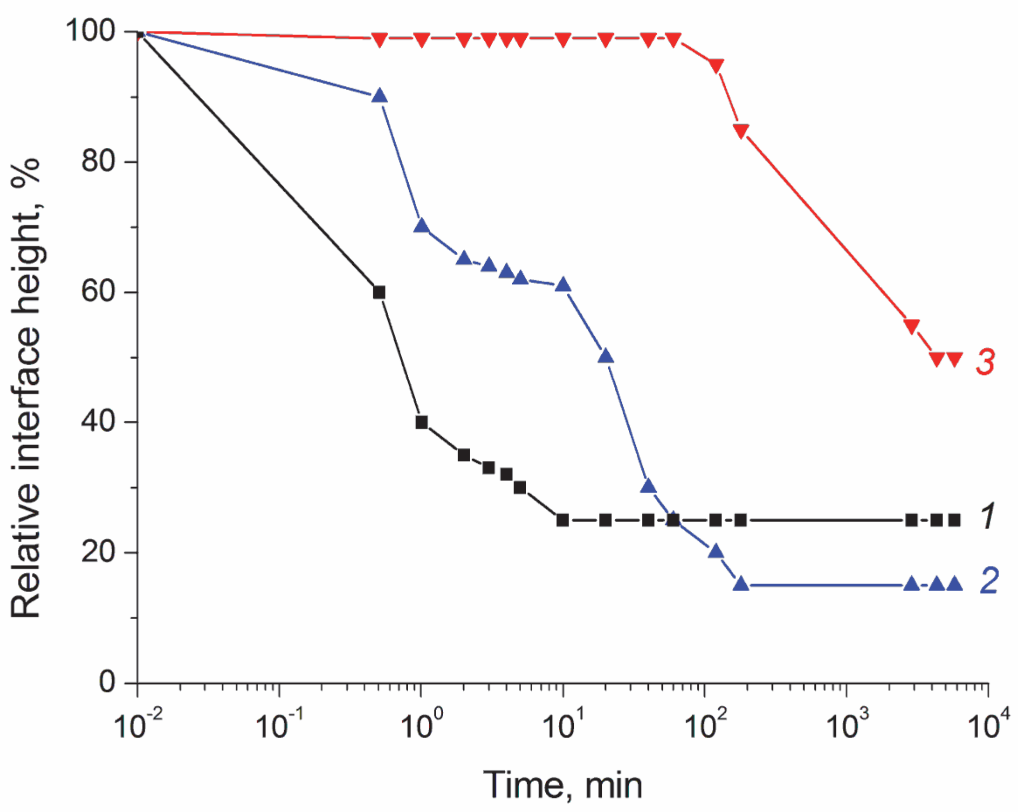

Fig. S2. Sedimentation curves for 10 wt.% suspensions of (*1*) WO_3, (*2*) WO_3/SDS and (*3*) WO_3/DDA powders in PMS-300 silicone oil.
